# Antifungal Activity of *Thymus vulgaris* L. Essential Oil and Its Constituent Phytochemicals against *Rhizopus oryzae*: Interaction with Ergosterol

**DOI:** 10.3390/molecules171214418

**Published:** 2012-12-05

**Authors:** Kelly Samara de Lira Mota, Fillipe de Oliveira Pereira, Wylly Araújo de Oliveira, Igara Oliveira Lima, Edeltrudes de Oliveira Lima

**Affiliations:** 1 Laboratory of Mycology, Department of Pharmaceutical Sciences, Federal University of Paraíba, João Pessoa, 58051-970, Brazil; E-Mails: igaralima@yahoo.com.br (I.O.L); edelolima@ltf.ufpb.br (E.O.L.); 2 Center of Education and Health, Federal University of Campina Grande, Cuité, 58175-000, Brazil; E-Mails: fillipeopereira@ufcg.edu.br (F.O.P.); wyllybr@yahoo.com.br (W.A.O.)

**Keywords:** *Thymus vulgaris*, thymol, *Rhizopus oryzae*, antifungal activity, essential oils

## Abstract

Mucormycoses are emerging infections that have high rates of morbidity and mortality. They show high resistance to antifungal agents, and there is a limited therapeutic arsenal currently available, therefore, there is a great need to give priority to testing therapeutic agents for the treatment of mucormycosis. Along this line, the use of essential oils and phytoconstituents has been emphasized as a new therapeutic approach. The objective of this work was to investigate the antifungal activity of the essential oil (EO) of *Thymus vulgaris*, and its constituents thymol and *p*-cymene against *Rhizopus oryzae*, through microbiological screening, determination of minimal inhibitory concentration (MICs) and minimal fungicidal concentration (MFCs), effects on mycelial growth and germination of sporangiospores and interaction with ergosterol. The MIC of EO and thymol varied 128–512 µg/mL, but the MFC of EO and thymol varied 512–1024 µg/mL and 128–1024 µg/mL, respectively. The results also showed that EO and thymol significantly inhibited mycelial development and germination of sporangiospores. Investigation of the mechanism of antifungal action showed that EO and thymol interact with ergosterol. These data indicate that EO of *T. vulgaris* and thymol possess strong antifungal activity, which can be related to their interaction with ergosterol, supporting the possible use of these products in the treatment of mucormycosis.

## 1. Introduction

Fungi have become increasingly recognized as important pathogens in critically ill patients. During the last decade, fungal infections, mainly those caused by opportunistic microorganisms, have been a problem of growing clinical importance [1,2,3]. In the literature, the incidence of opportunistic infections in hospital environments or nosocomial infections is related to the fungi belonging to the genera *Candida*, *Aspergillus*, *Rhizopus*, *Penicillium*, *Fusarium* and *Cryptococcus*, among others [4,5]. There has been a significant increase in infections due to emerging fungi, such as *Scedosporium*, *Fusarium* and various zygomycetes, including *Mucor* and especially *Rhizopus* [1,2]. 

Zygomycoses are infections caused by fungi of the class Zygomycetes, particularly the filamentous fungi belonging to two orders of clinical importance, which cause infections in humans, *i.e.*, the Mucorales and Entomophthorales [6]. However, the majority of human diseases are caused by the order Mucorales, and are thus known as mucormycoses. Members of this order have been increasingly capable of causing opportunistic infections that are progressive, necrotic and generally fatal, in a variety of immunocompromised hosts, such as patients with hematological diseases, with neutropenia, on corticosteroids, with diabetes mellitus with or without ketoacidosis, or with solid organ transplants, and patients with high levels of serum iron. These pathogens rarely cause infections in immunocompetent patients [6,7,8]. 

The incidence of murcormycosis has increased in the last years, representing the third most common invasive fungal infection, after candidiasis and aspergillosis [9,10]. It is considered an increasingly emerging and potentially fatal infection, due to its high levels of morbidity and mortality [1,10,11]. The major forms of clinical manifestations of zygomycosis (mucormycosis) include rhinocerebral, pulmonary, cutaneous, gastrointestinal and disseminated or systemic symptoms [12]. The most common organisms that cause zygomycosis in humans are of the genera *Rhizopus*, *Mucor*, *Rhizomucor* and *Absidia*. *Rhizopus* stands out the most because many of its species cause more than 70% of infections by Zygomycetes, where *R. oryzae* is the most common etiological agent [7,13].

The standard therapy for invasive zygomycosis consists in the reversal of underlying predisposing factors, surgical intervention such as aggressive debridement or amputation and drug treatment [8,12]. Traditionally, amphotericin B and more recently its lipid formulations comprise the first line treatment of zygomycosis [14]. Specifically, liposomal amphotericin B is less nephrotoxic and shows better penetration of the central nervous system compared to amphotericin B and other lipid formulations [15]. The therapeutic arsenal for the treatment of mucormycosis is limited, plus there are various side effects associated with the use of these antifungal agents. Moreover, since many patients who develop these aggressive fungal infections show high mortality rates, there is a tremendous necessity for new therapeutic strategies aimed at introducing into the pharmaceutical market new products, natural or synthetic, for the treatment of mucormycosis. Natural products have been especially considered as sources of potentially bioactive molecules, which may have a wide spectrum of actions and fewer collateral effects. 

The natural products that have stirred great interest in the scientific community are essential oils, which are complex mixtures rich in terpenes with different degrees of lipophilicity and relative hydrophilicity [16]. Such compounds can alter cell permeability by their insertion between the fatty acid chains that compose the lipid bilayers of membranes, thereby interrupting lipid packing, causing alterations in the properties and functions of the cell membrane by increasing its fluidity and permeability [17,18]. Essential oils and their phytoconstituents have shown promising antifungal activity *in vitro* and *in vivo*, where they have been extensively studied against *Candida* spp., *Trichophyton* spp. and *Aspergillus* spp. [18,19,20,21,22,23,24,25,26,27]. However, studies on the antifungal activity of essential oils and their components against species of *Rhizopus*, the main genus responsible for causing the majority of mucormycosis, are scarce.

In view of these considerations, the aim of the present work was to investigate the antifungal activity of 10 essential oils, choosing among them that which showed the best antifungal profile for an in-depth study of its fungicidal and/or fungistatic effects against *R. oryzae* strains, of its effects on mycelial growth and germination of sporangiospores, *R. oryzae* and of its mechanism of action as well.

## 2. Results and Discussion

During the last decade, advances in diagnostic systems and the introduction of new antifungal agents significantly improved the prognosis of patients who develop invasive fungal infections, mainly those who are immunocompromised. However, morbidity and mortality rates remain relatively high for mucormycosis, the third most common invasive fungal infection after candidiasis and aspergillosis [1,10,11,14]. The resistance of causative microorganisms has been a concern and has gained great clinical importance, since many zygomycetes are resistant to the majority of antifungals that are utilized to treat systemic mycosis, including flucytosine or 5-fluorocytosine (5-FC), ketoconazole, fluconazole, voriconazole, and the echinocandins. Furthermore, zygomycetes have variable sensitivity to itraconazole and terbinafine. However, the majority of these pathogenic fungi are sensitive to amphotericin B and pasaconazole, a new triazole antifungal [28,29,30]. Accordingly, a disk diffusion assay in solid medium was carried out to evaluate the sensitivity of *R. oryzae* to some antifungals currently on the pharmaceutical market. All strains tested were found to be resistant to the five antifungal drugs evaluated, namely amphotericin B, itraconazole, fluconazole, 5-fluorocytosine and miconazole, since they showed no inhibition zone or one with a diameter ≤ 10 mm (Table 1). 

The results obtained reinforce the importance and necessity of research on the potential use of essential oils as a new therapeutic alternative in the treatment of fungal infections, due to emerging drug resistance, mainly related to zygomycetes. These findings prompted the investigation of the antifungal profile of 10 essential oils against different resistant strains of *R. oryzae* by microbiological screening. The crude essential oils of *Coriandrum sativum*, *Hyptis suaveolens* and *Origanum majorana*,did not show antifungal activity, since the diameters of the inhibition zones were ≤10 mm. However, the essential oils of *Ocimum basilicum*, *Cymbopogon citratus*, *C. martini*, *C. winterianus*, *Cinnamomun zeylanicum*, *Thymus vulgaris* and *Origanum vulgare* showed a strong and wide spectrum of antifungal action, with mean inhibition zones of 13–32 mm. *O. vulgare and T. vulgaris* displayed the best activity, both with mean inhibition zones of 32 mm (Table 2). In the last years, a large number of essential oils, especially those of some species of *Thymus* and their phenolic components have been investigated for their antimicrobial properties against certain bacteria [31], protozoans [32] and fungi [33,34,35,36], although there still exist a little information in the literature about the possible mechanisms of action of *T. vulgaris* essential oil and its components Therefore, considering the important antimicrobial potential of the genus *Thymus*, together with evidence that the essential oil of *T. vulgaris* shows one of the best antifungal profiles in fungal susceptibility tests using the disk diffusion assay in solid medium, this essential oil was chosen for further study with the aim of elucidating its antifungal mode of action.

**Table 1:** Susceptibility of *R. oryzae* strains to antifungal drugs.

Fungal strain	Sensitivity to antifungal drugs (diameter of inhibition zone in mm)				
	AMB	ICZ	FLU	5-FC	MCZ
LM-03	0	0	0	0	0
LM-04	10	0	0	0	0
LM-25	7	0	0	0	0
LM-29	0	0	0	0	0
LM-508	10	0	0	0	0
LM-766	0	0	0	0	0
LM-810	0	0	0	0	0
RO-5786	0	0	0	0	0
RO-4692	0	0	0	0	0
RO-4565	0	0	0	0	0
RO-4557	0	0	0	0	0

All experiments were performed in duplicate. Amphotericin B (AMB—100 μg disk^−1^), itraconazole (ICZ—10 μg disk^−1^), fluconazole (FLU—25 μg disk^−1^), 5 fluorocytosine (5-FC—10 μg disk^−1^) and miconazole (MCZ—50 μg disk^−1^).

**Table 2:** Antifungal activity of essential oils against strains of *R. oryzae.*

Essential oil	Sensitivity to essential oils (diameter of inhibition zone in mm)						
	LM-03	LM-04	LM-28	LM-29	LM-508	LM-766	LM-810
*C. citratus*	25	18	28	23	24	0	24
*C. martini*	17	16	17	16	18	20	0
*C. winterianus*	16	17	18	16	22	20	24
*T. vulgaris*	29	32	28	28	31	38	36
*C. zeylanicum*	24	23	23	26	25	26	27
*O. vulgare*	33	33	31	31	30	38	30
*O. basilicum*	15	16	13	0	0	0	0
*C. sativum*	0	0	0	0	0	0	0
*H. suaveolens*	0	0	0	0	0	0	0
*O. majorana*	0	0	0	0	0	0	0

All experiments were performed in duplicate. Each disk contained 10 μL of essential oils.

Table 3 summarizes the minimum inhibitory concentrations (MICs) and minimum fungicidal concentrations (MFCs) of the drugs tested. The MIC of the essential oil was 256 µg/mL for 83% of the strains evaluated. However, for the main phytoconstituents, thymol and *p-*cymene, the MICs were 128 µg/mL (83% of strains) and >1,024 µg/mL (100% of strains), respectively. The MIC for the positive control amphotericin B was 4 µg/mL for 92% of the strains investigated. All fungal strains were capable of growing in the absence of the products, which demonstrated their viability (microorganism control). These results indicate that *p*-cymene did not show significant inhibition of growth against strains of *R*. *oryzae*. Meanwhile, the essential oil of *T. vulgaris* and thymol displayed strong antifungal activity, where thymol was twice as potent, when compared to this complex mixture, thus confirming its antifungal potential, since essential oils with a MIC between 50 and 500 μg/mL are considered to have strong antimicrobial activity, while MICs between 600 and 1,500 μg/mL and over 1,500 μg/mL indicate moderate and weak activity, respectively [37]. These are important results, confirmed in the Table 4 which shows that the essential oil of *T. vulgaris* contain 46.6% of thymol. This indicates that the antifungal effect is probably the result of thymol only.

After determination of the MIC, the fungicidal effect of the products was investigated. The MFC values of the essential oil and thymol varied 512–1024 µg/mL and 128–1,024 µg/mL, respectively (Table 3), where the MFCs of the essential oil corresponded to 2 × MIC or 4 × MIC for the majority of the *R. oryzae* strains. MFC of thymol was equal to MIC or 2 × MIC for majority of the *R. oryzae* strains *R. oryzae*. Comparing the MFC values of the majority of strains tested, thymol exhibited approximately two to four times more potent fungicidal activity compared to the essential oil of *T. vulgaris*.

**Table 3:** Minimal inhibitory concentrations (MIC) and minimum fungicidal concentrations (MFC).

*R. oryzae*	Essential oil (µg/mL)		Thymol (µg/mL)		*p*-Cymene (µg/mL)	AMB (µg/mL)
	MIC	MFC	MIC	MFC	MIC	MIC
LM-03	256	512	128	128	>1024	4
LM-04	256	ND	128	ND	>1024	4
LM-25	256	1024	128	256	>1024	4
LM-28	256	1024	128	256	>1024	4
LM-29	256	1024	128	256	>1024	4
LM-508	256	>1024	128	128	>1024	4
LM-766	256	512	128	256	>1024	4
LM-810	256	1024	128	256	>1024	4
RO-5786	512	1024	256	256	>1024	4
RO-4692	512	1024	256	1024	>1024	4
RO-4565	256	>1024	128	512	>1024	2
RO-4557	256	1024	128	256	>1024	4

Earlier studies demonstrated that the essential oils of *Thymus spp.* display a wide spectrum of fungicidal and/or fungistatic activity. The essential oils of *T. eriocalyx* and *T. x-porlock*, whose major phytoconstituent is thymol (64.3 and 30.7%, respectively) exhibited strong fungistatic and fungicidal activities against *Aspergillus parasiticus* [34]. In addition, the essential oil of *T. spathulifolius*, whose thymol content is 36.5%, inhibited the growth of *Trichophyton spp*., *Fusarium spp*., *Penicillium spp*., *Rhizopus spp*., *Alternaria spp*. and *Aspergillus spp*., with MICs varying between 31 and 250 µg/mL [38]. Giordani *et al.* [39] carried out a study on the antifungal potential of essential oils of various chemotypes of *T. vulgaris* against *Candida albicans.* The essential oil of the thymol chemotype of *T. vulgaris* was the most potent, with a MIC_80%_ of 0.016 µL/mL, where the efficacy was mainly due to the high level of thymol (63.2%). According to Klaric *et al.* [36], both thymol and the essential oil of *T. vulgaris*, whose main components are *p*-cymene (36.5%) and thymol (33.0%) showed strong fungicidal and/or fungistatic activities against *Aspergillus*, *Penicillium*, *Cladosporium*, *Trichoderma*, *Mucor* and *Rhizopus*. Thymol exhibited three times greater inhibition compared with the essential oil of *T. vulgaris*.

Many investigators have demonstrated the antifungal potential of thymol against species of yeasts and filamentous fungi. Thymol inhibits the growth of *Candida* species sensitive and resistant (clinical isolates) to azoles and amphotericin B [27,33,40], and interferes with the formation and viability of hyphae of *C. albicans* [18]. Similar results were reported for *Aspergillus fumigatus* and *Trichophyton rubrum* resistant to azoles and amphotericin B [26]. However, there are few studies on the antifungal activity of *p*-cymene. *p-*Cymene and 1,8-cineol have been found to be much less effective against *Aspergillus spp*. and *Penicillium spp*., (MIC ≥ 4 or 8%, v/v), when compared with thymol [41]. However, with regard to opportunistic yeasts, thymol and *p*-cymene, alone or in combination, exhibit strong antifungal activity against *Candida spp*. [33]. 

Comparing the phytochemical profiles of the essential oils of various species of *Thymus* with that of the thymol chemotype of *T. vulgaris* used in the present study (Table 4), shows that the principal components of the majority of *Thymus* species are *p*-cymene and thymol. 

**Table 4:** Chemical composition of *T. vulgaris* essential oil.

Constituent	%
α-Pinene	3.3
Camphene	1.0
β-Pinene	0.6
Myrcene	1.7
*p*-Cymene	38.9
Limonene	0.8
1,8-Cineole	1.2
γ-Terpinene	0.3
Linalool	3.8
Thymol	46.6

Peaks less than 0.1% were excluded.

According to Kalemba and Kunicka [42], antifungal activity can be classified in the following decreasing order: phenols > aldehydes > ketones > alcohols > esters > hydrocarbons. Therefore, in agreement with these observations, it is evident that there is a relation between the strong activity of *Thymus* essential oil and the high percentage of phenolic components, such as thymol. Correlating structure with activity, it can be speculated that the fungicidal and/or fungistatic activity of the essential oil of *T. vulgaris* can be attributed to thymol, its principal constituent, especially the hydroxyl group of this compound, since *p*-cymene (benzene), the second major component, does not possess substantial antifungal activity. This would explain the lower potency of the oil when compared to thymol, supporting the idea that the efficacy of essential oils depends on its chemical composition, mainly phenolic components. These results are of great importance, because they facilitate the utilization of individual components, instead of a mixture, giving more predictability and probably less collateral effects.

**Figure 1:**
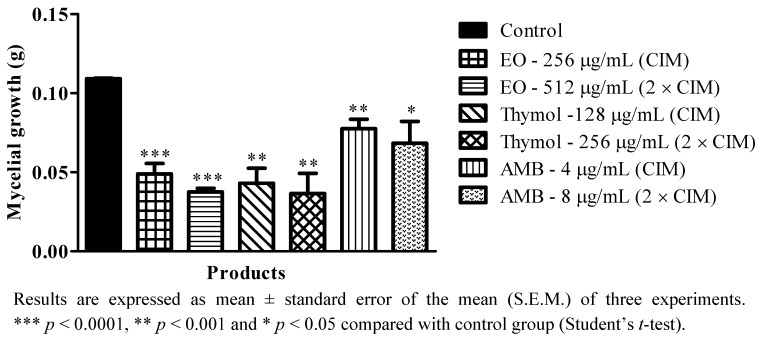
Effect of *T. vulgaris* essential oil, thymol and amphotericin B on mycelial growth of RO-4557.

Macromolecules whose functionality is related to growth, survival, virulence or cellular morphogenesis are pointed out as promising targets for new antifungal agents [43]. Thus, taking into consideration the promising antifungal activity of the oil essential of *T. vulgaris* and of its major phytoconstituent, thymol, the effect of different concentrations of these substances on mycelial growth and the germination of sporangiospores of *R. oryzae* (RO-4557) was investigated. The results obtained demonstrated that the essential oil, thymol and amphotericin B at concentrations equal to MIC and 2 × MIC significantly reduced the dry mycelial mass of *R. oryzae* (Figure 1), with the following percentages of inhibition: 55 ± 6% (MIC of EO), 66 ± 2% (2 × MIC of EO), 61±9% (MIC of thymol), 67 ± 12% (2 × MIC of thymol), 29 ± 5% (MIC of amphotericin B) and 37 ± 13% (2 × MIC of amphotericin B) when compared with the normal control of RO-4557. These results suggested that the substances evaluated inhibited normal mycelial development of *R. oryzae* at all concentrations tested. These results corroborate the data obtained by some researchers who have investigated the antifungal potential of essential oils in inhibiting the mycelial growth of pathogenic and non-pathogenic fungi [24,34]. The essential oils of two varieties of thyme, *Thymus eriocalyx* and *Thymus x*-*porlock*, inhibited the mycelial growth of *Aspergillus parasiticus* [34]. Recently, our research group showed that the essential oil of *C. winterianus* inhibited the mycelial development of *Trichophyton mentagrophytes* [24]. 

**Figure 2:**
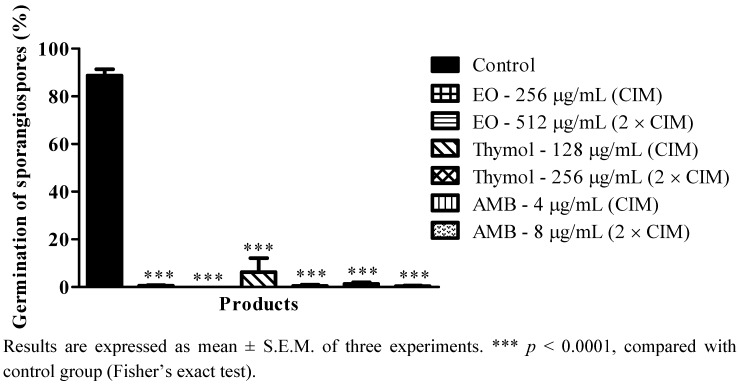
Effect of *T. vulgaris* essential oil, thymol and amphotericin B on the germination of sporangiospores of RO-4557.

The results reported to date can be considered of great relevance, due to the importance of mycelial growth in the development of mucormycosis, since the fungi of the order Mucorales, including the species of *Rhizopus*, the main causative agent of these infections, are characterized by an erect aerial mycelium, described as fibers or “cotton candy”, which grow well and rapidly [7]. These fungi cause extensive angioinvasion, which is a striking characteristic, infiltrating through the blood vessels, resulting in thrombosis of the vessels and tissue necrosis [1,44]. Mucormycosis, fungal infections that are often fatal, begin by contact with the host through aerial sporangiospores, which initiate the infectious process with the germination of sporangiospores and formation of mycelium. Thus, the study of the germination of sporangiospores has great implications in clinical practice, because it is possible to develop new therapeutic approaches that block the infection at its onset [45]. In this perspective, the effect of the essential oil of *T. vulgaris* and its principal phenolic component on the germination of the sporangiospores of RO–4557 was investigated. The effects of different concentrations (MIC and 2 × MIC) of *T. vulgaris* essential oil, thymol and amphotericin B on the germination of sporangiospores are shown in Figure 2. At all concentrations tested, the essential oil, thymol and amphotericin B exerted a strong inhibitory effect on the germination process of the sporangiospores of *R. oryzae*, where the percentage of inhibition varied between 94 and 100%. 

The antifungal potential of essential oils in inhibiting the germination of sporangiospores has been extensively studied. It has been reported that the essential oil of *C. winterianus* has a strong inhibitory effect on the germination of conidia of *T. mentagrophytes* [24]. The essential oil of *C. zeylanicum* was shown to inhibit the germination of the conidia of *A. fumigatus*, *A. flavus* and *A. niger* [46]. Also, in view of the importance of the germination of sporangiospores of *Rhizopus* as the primary cause of zygomycosis, some studies have reported on the inhibitory effect of certain drugs on this process. Lovastatin produces a significant delay in the germination of sporangiospores of *Rhizomucor pusillus* [47]. Additionally, statins (lovastatin, sinvastatin, rosuvastatin and atorvastatin) were reported to inhibit the germination of sporangiospores of fungi of the class of zygomycetes [48]. It has been found that *N*-acetyl-cysteine and its derivatives inhibit the germination of sporangiospores of different zygomycetes, especially *R. oryzae* [49]. The results obtained in this study corroborate those observed in previous studies, thus revealing the antifungal potential of the essential oil and thymol in blocking the infection induced by *R. oryzae* soon after onset, since they significantly inhibit the germination of sporangiospores.

Considering the lipophilic nature of essential oils and of their phenolic components, as well as the interaction of these products with biological membranes, it was decided to investigate the participation of membrane sterols in the antifungal effect exerted by the essential oil of *T. vulgaris* and thymol. Ergosterol is the principal sterol present in yeasts and filamentous fungi, where it is necessary for the growth and normal function of the fungal cell membrane. Besides controlling the fluidity, asymmetry and integrity of the membrane, ergosterol contributes to the proper functioning of enzymes bound to the membrane [50]. The majority of existing drugs for the treatment of fungal infections target the cell wall or plasma membrane directly or indirectly, particularly ergosterol and its biosynthesis [43,50]. Therefore, an interaction assay was performed with ergosterol, present only in fungal membranes, and cholesterol, present in the cell membrane of mammals. This method is based on the exposure of a test compound to exogenous sterols, where an affinity for sterols will lead to the rapid formation of a complex, thereby impeding complexation with sterols of the membrane and resulting in an increase in MIC [51]. The MICs of the essential oil and thymol against *R. oryzae* increased four and eight times in the presence of 200 and 400 µg/mL ergosterol, respectively. With cholesterol, the MICs of the drugs increased fourfold for both concentrations. Amphotericin B, the positive control that has a known interaction with ergosterol, showed a 256-fold higher MIC in the presence of this sterol. A similar interaction was seen in the presence of cholesterol (Table 5).

**Table 5:** Effect of EO of *T. vulgaris*, thymol and amphotericin B against *R. oryzae* (RO-4557) in absence and presence of sterols.

Drug	MIC (µg/mL)				
	Absence of sterols	Presence of ergosterol		Presence of cholesterol	
		200 µg/mL	400 µg/mL	200 µg/mL	400 µg/mL
EO	256	1024	2048	1024	1024
Thymol	128	512	1024	512	512
AMB	4	1024	1024	512	1024

All experiments were performed in triplicate.

The action of essential oils and their phenolic components on the cell membrane has been widely studied. The essential oils of *Thymus spp*., especially *T. zygis* and *T. vulgaris* and their components, such as carvacrol, thymol and *p*-cymene, have displayed potent fungicidal activity against *Candida spp*., resulting mainly in extensive damage to the cell membrane [33]. The essential oils of *T. eriocalyx* and *T. x-porlock* have been shown to cause irreversible damage to the cell wall, organelles and cell membrane of *A. parasiticus* [52]. There are few studies on the direct interaction of essential oils and their phytoconstituents with ergosterol of the membrane, although there is a large variety of biologically active compounds, such as polyenes, whose main representative is amphotericin B, which binds directly to ergosterol and forms pores that destabilize the membrane, resulting in eventual loss of intracellular material and cell lysis [43,53].

The results obtained suggest that the mechanism of the antifungal action of the essential oil and thymol involves a direct interaction with ergosterol, which leads to the disruption of the fungal membrane and loss of intracellular contents. However, such action is not selective, since there is also an interaction between the study products and cholesterol. This also holds true for amphotericin B, which has a amphipathic character, thus possessing the capacity to bind to both sterols incorporated in cell membranes, ergosterol and cholesterol, resulting in toxicity to mammalian cells and particularly causing nephrotoxicity [43,53,54]. 

## 3. Experimental

### 3.1. Plant Essential Oil and Drugs

Plant essential oils of *Cymbopogon citratus* (DC.) Stapf (capim santo), *Cinnamomum zeylanicum* Blume (canela), *Coriandrum sativum* L. (coentro), *Origanum majorana* L. (manjerona) and *Origanum vulgare* L. (oregano) were obtained from Ferquima Industria e Comercio Ltda. (Vargem Grande Paulista, São Paulo, Brazil); essential oils of *Cymbopogon martini* (palmorosa), *Ocimum basilicum* (manjericão) and *Thymus vulgaris* L. (tomilho) were obtained from Laszlo Aromaterapia Ltda. (Belo Horizonte, Minas Gerais, Brazil); *Cymbopogon winterianus* Jowitt ex Bor (citronela) and *Hyptis suaveolens* L. (alfazema brava) were obtained from the Experimental Plant Collection, Department of Agriculture, Center of Technologist Training, Federal University of Paraíba, Bananeiras, Brazil. The purity of oils was determined by percent composition of major active compounds as revealed by gas chromatography-mass spectrometry (GC-MS) (data not shown). The major compounds of *T. vulgaris*, thymol and *p*-cymene, and amphotericin B (positive control) were acquired from Sigma‑Aldrich^®^ (São Paulo, SP, Brazil). The essential oil of *T. vulgaris* and its phytoconstituents were dissolved in 2% Tween 80 (INLAB/Industria Brasileira, São Paulo, SP, Brazil) and amphotericin B in 1% dimethyl sulfoxide (DMSO) in sterile distilled water to obtain 1,024 µg/mL solutions.

### 3.2. Essential Oil Analysis

The composition of essential oils was analyzed using GC-MS on a Shimadzu GC-17A/MS QP5050A (GC/MS system) apparatus equipped with a HP-1 column (30 m × 0.25 mm id, 0.25 μm film thickness). Helium was employed as the carrier gas at a flow rate of 1.0 mL/min; column inlet pressure was 48.7 kPa; linear velocity = 36.0 cm/s; total flow rate was 50 mL/min; carrier flow rate was 24 mL/min; injector temperature was 250 °C; detector temperature was 250 °C; column temperature was 40 (3 min)–150 °C (1 min) at 3 °C/min, then 150–250 °C at 10 °C/min (10 min). For GC-MS detection an electron ionization system was used with ionization energy of 70 eV. Samples were diluted 1/1000 (v/v) in hexane and 1.0 µL were injected in the splitless mode [55]. The compounds were identified by comparing their fragmentation patterns detected in the mass spectra with those in the NIST 98 mass spectrometry library (National Institute of Standards and Technology, Gaithersburg, MD, USA) and with reports from the literature. The quantification of the components was based on the percentage of peak area of each component in relation to the total area of all standardized peaks in the chromatogram.

### 3.3. Mold Strains

In the antifungal tests, we used eight strains of *R. oryzae* (LM-03, LM-04, LM-25, LM-28, LM-29, LM-508, LM-766 and LM-810), which were isolated, identified and stored in the Mycology Laboratory of the Department of Pharmaceutical Sciences, Center of Health Sciences, Federal University of Paraiba, and four strains of *R. oryzae* (RO-4557, RO-4565, RO-4692 and RO-5786) obtained from the Culture Collection—URM, of the Department of Mycology, Federal University of Pernambuco, Recife, PE, Brazil. The fungi were stored on potato dextrose agar (PDA—Difco Laboratory, Detroit, MI, USA) at 4 °C until used in tests.

### 3.4. Inoculum

The inoculum preparation was adapted from Dannaoui *et al.* [29] and Espinel-Ingroff *et al.* [56]. The fungi were grown at 28 °C on Sabouraud dextrose agar (SDA) (Difco) until they were judged to have formed maximal numbers of sporangiospores (5 days). The stock sporangiospore suspensions were prepared by washing the surface of the slants with 5 mL of sterile saline and shaking suspensions for 5 min. The resulting mixture of sporangiospores and hyphal fragments was withdrawn and transferred to a sterile tube. After heavy particles were allowed to settle for 3 to 5 min, the upper homogeneous suspension was collected and vortexed for 15 s. The resulting sporangiospore suspension was counted with a hemocytometer, where it was adjusted to 10^6^ sporangiospores/mL.

### 3.5. Disk Diffusion Assay

The disk diffusion assay was performed to determine the sensitivity of fungal strains to the antifungal drugs and essential oils [57,58]. Briefly, 1 mL of spore suspension (10^6^ sporangiospores/mL) was spread onto SDA plates and filter paper discs (Sensiobiodisc, CECON/SP) impregnated with 10 μL of essential oils, whereas for drug sensitivity, antifungal drug disks (10–100 μg/disk, CECON/SP), were mounted on the agar surface and the plates were incubated at 28 ± 2 °C for 2 days. Each experiment was conducted in duplicate and average zone size was measured. The antifungal activity of the products was considered positive when the arithmetic mean was greater than or equal to 10 mm of at least 50% of all strains tested. The essential oil with the best antifungal profile was chosen to characterize the antifungal activity *in vitro*.

### 3.6. Determination of MIC and MFC

The broth microdilution assay with some modifications as adapted by Dannaoui *et al.* [29] and Espinel‑Ingroff *et al.* [56] was performed to determine the MIC of *T. vulgaris* essential oil, thymol, *p*‑cymene and amphotericin B against *R. oryzae*. On the day of the test, sterile 96-U-shaped-well microplates were used and each well of the plates contained 100 μL of Sabouraud dextrose broth (SDB) (Difco Lab.). Afterwards, 100 µL of the products (1,024 µg/mL) were added to the first wells. Next, serial twofold dilutions in culture medium were prepared to obtain concentrations ranging from 0.25 to 1,024 µg/mL. Finally, 10 μL of fungal inoculum were added to all wells. The microplates were incubated at 28 °C and MICs were determined visually after 48 h incubation. The MIC was determined from three independent experiments and was defined as the lowest drug concentration that showed absence of growth or complete fungal growth inhibition (100% inhibition). Negative control (without drugs) was performed to confirm the viability of the sporangiospores. Sensitivity control for Tween 80 and DMSO was also performed. The MFC was determined for the drugs that showed strong antifungal activity. After determining the MIC, 10 μL were subcultured from each well that showed complete inhibition (100% or an optically clear well) on SDA plates. The plates were incubated at 28 °C for 24 h, and the MFC was the lowest thyme and thymol concentration that showed either no growth or fewer than three colonies to obtain approximately 99 to 99.5% killing activity. The MFC was determined from three independent experiments on different occasions.

### 3.7. Effects on Mycelial Growth

The analysis of the interference of *T. vulgaris* essential oil, thymol and amphotericin B on mycelial growth was performed by determining the dry mycelial weight of *R. oryzae* 4557 [59,60]. Sterile tubes containing 4.5 mL of the drugs at concentrations corresponding to the MIC and 2 × MIC in SDB, were inoculated with 0.5 mL of a suspension of 10^6^ sporangiospores/mL. A control experiment was performed with sterile distilled water instead of the drugs. The tubes were incubated at 28 °C for five days. Cultures were filtered through sterile filter paper (retention of particles: 11 µm). The mycelia were dried at 60 °C for 10 min. The filter paper containing dry mycelium was weighed and the dry mycelium weight was expressed in grams for three independent experiments.

### 3.8. Sporangiospore Germination Assay

Sporangiospore germination was performed according to Sharma and Tripathi [60] and Shiosaki *et al.* [61] with some modifications. The essential oil, thymol and amphotericin B (positive control) were tested to evaluate the effectiveness of these products on inhibiting the germination of *R. oryzae* (RO-4557). A negative control was performed. Doubly concentrated SDB (500 µL) containing the drugs at concentrations corresponding to the MIC and 2 × MIC was added to sterile tubes. They were mixed with 500 µL of fungal suspension of 10^6^ sporangiospores/mL and immediately incubated at 28 °C. Samples were taken at 24 h for analysis. The number of germinated and ungerminated sporangiospores was determined in a hemocytometer and the percentage of sporangiospores germinated was determined. The test was performed in three independent experiments.

### 3.9. Membrane Sterols Assay

To determine if *T. vulgaris* L. essential oil and thymol interacts with ergosterol, the MIC of the products against *R. oryzae* (RO-4557) was determined by the microdilution method previously described, in the presence and absence of different concentrations (200 and 400 μg/mL) of ergosterol and cholesterol (Sigma-Aldrich^®^, São Paulo, SP, Brazil). Amphotericin B was used as the control drug for ergosterol tests. MIC was determined after 5 days. This assay was performed in triplicate [51].

### 3.10. Statistical Analysis

The results are expressed as mean ± S.E.M. Differences between the means were statistically compared using Student’s t-test or Fisher’s exact test. The values were considered significantly different when *p* < 0.05.

## 4. Conclusions

On the basis of the data presented, the essential oil of *T. vulgaris* and its phenolic component, thymol, have promising fungicidal and/or fungistatic activity, whereby they are capable of inhibiting an infection at its onset. Such activities can be related to an interaction with ergosterol, a sterol present in the cell membrane of *R. oryzae*, which plays an important role in the growth and normal function of the cell membrane of these fungi. Therefore, these thyme products, especially thymol, may represent new alternative therapeutic agents in the treatment of mucormycosis. However, there is a need for more studies aimed at correlating their potent antifungal activity *in vitro* and *in vivo* and proving their safety for clinical application.
